# Supplementary material for: Laser-Synthesized Elemental Boron Nanoparticles for Efficient Boron Neutron Capture Therapy
Source: Int J Mol Sci. 2023 Dec 4;24(23):17088. doi: 10.3390/ijms242317088 (PMC10707216; doi:10.3390/ijms242317088)
Supplement: Supplementary file 1 [file ijms-24-17088-s001.zip › ijms-2699135-supplementary.pdf]

Supplementary Materials

Laser-synthesized elemental boron nanoparticles for efficient boron neutron capture therapy

Irina N. Zavestovskaya, Anna I. Kasatova, Dmitry A. Kasatov, Julia S. Babkova, Ivan V. Zelepukin, Ksenya S. Kuzmina, Gleb V. Tikhonowski, Andrei I. Pastukhov, Kuder O. Aiyyzhy, Ekaterina V. Barmina, Anton A. Popov, Ivan A. Razumov, Evgenii L. Zavjalov, Maria S. Grigoryeva, Sergey M. Klimentov, Vladimir A. Ryabov, Sergey M. Deyev, Sergey Yu. Taskaev, and Andrei V. Kabashin

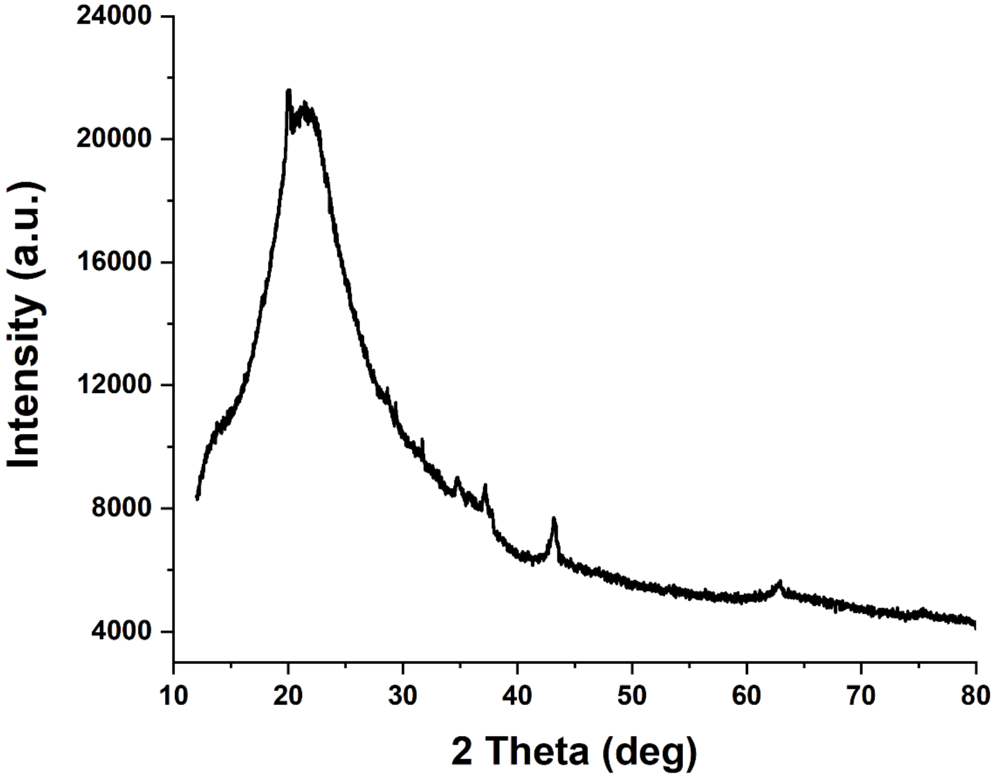

Figure S1. X-Ray diffraction pattern for the powder of a-BNPs, obtained by ns laser ablation.

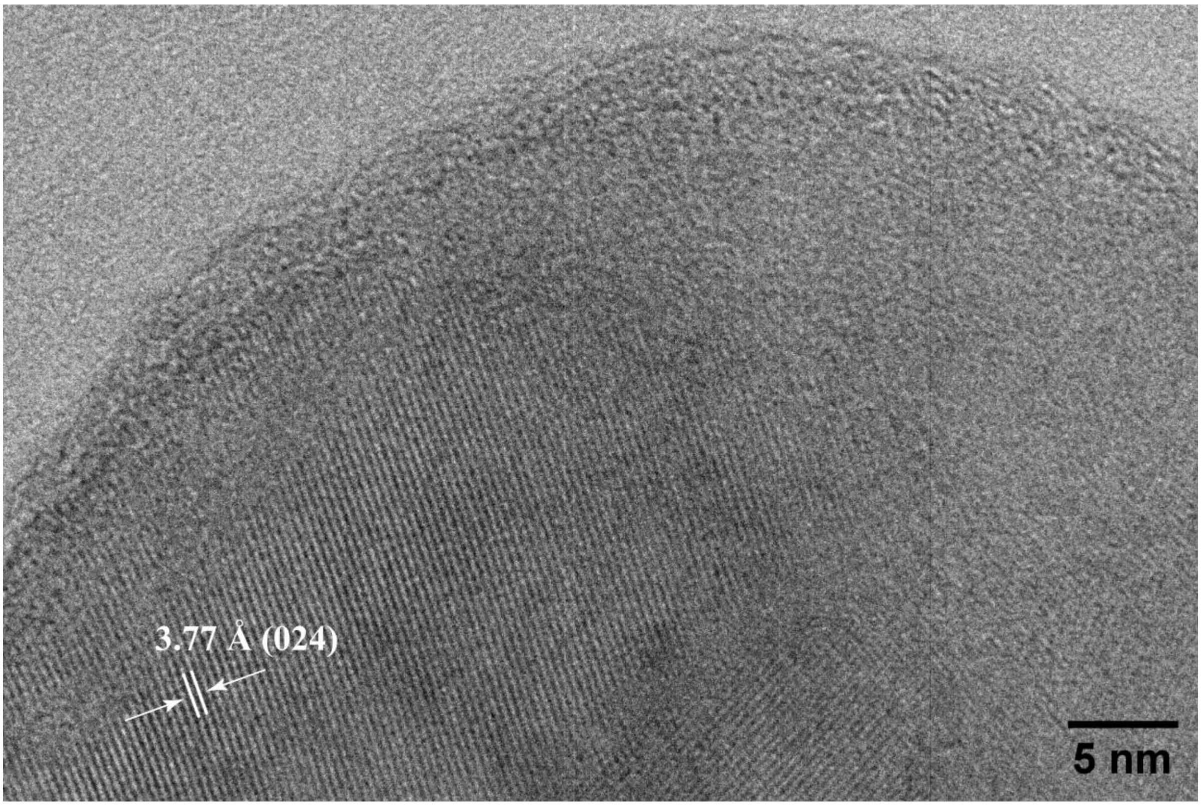

Figure S2. HR-TEM image of partial crystalline pc-BNPS, obtained by fs laser ablation. Scale bar - 5 nm.
